# The Trichohyalin-Like Protein Scaffoldin Is Expressed in the Multilayered Periderm during Development of Avian Beak and Egg Tooth

**DOI:** 10.3390/genes12020248

**Published:** 2021-02-10

**Authors:** Veronika Mlitz, Marcela Hermann, Maria Buchberger, Erwin Tschachler, Leopold Eckhart

**Affiliations:** 1 Skin Biology Laboratory, Department of Dermatology, Medical University of Vienna, 1090 Vienna, Austria; veronika.mlitz@meduniwien.ac.at (V.M.); maria.buchberger@meduniwien.ac.at (M.B.); erwin.tschachler@meduniwien.ac.at (E.T.); 2 Department of Medical Biochemistry, Medical University of Vienna, 1090 Vienna, Austria; marcela.hermann@meduniwien.ac.at

**Keywords:** keratinocytes, epidermis, trichohyalin, birds, beak, rhamphotheca, periderm, egg, development, evolution

## Abstract

Scaffoldin, an S100 fused-type protein (SFTP) with high amino acid sequence similarity to the mammalian hair follicle protein trichohyalin, has been identified in reptiles and birds, but its functions are not yet fully understood. Here, we investigated the expression pattern of scaffoldin and cornulin, a related SFTP, in the developing beaks of birds. We determined the mRNA levels of both SFTPs by reverse transcription polymerase chain reaction (RT-PCR) in the beak and other ectodermal tissues of chicken (*Gallus gallus*) and quail (*Coturnix japonica*) embryos. Immunohistochemical staining was performed to localize scaffoldin in tissues. Scaffoldin and cornulin were expressed in the beak and, at lower levels, in other embryonic tissues of both chickens and quails. Immunohistochemistry revealed scaffoldin in the peridermal compartment of the egg tooth, a transitory cornified protuberance (caruncle) on the upper beak which breaks the eggshell during hatching. Furthermore, scaffoldin marked a multilayered peridermal structure on the lower beak. The results of this study suggest that scaffoldin plays an evolutionarily conserved role in the development of the avian beak with a particular function in the morphogenesis of the egg tooth.

## 1. Introduction

**Figure 1:**
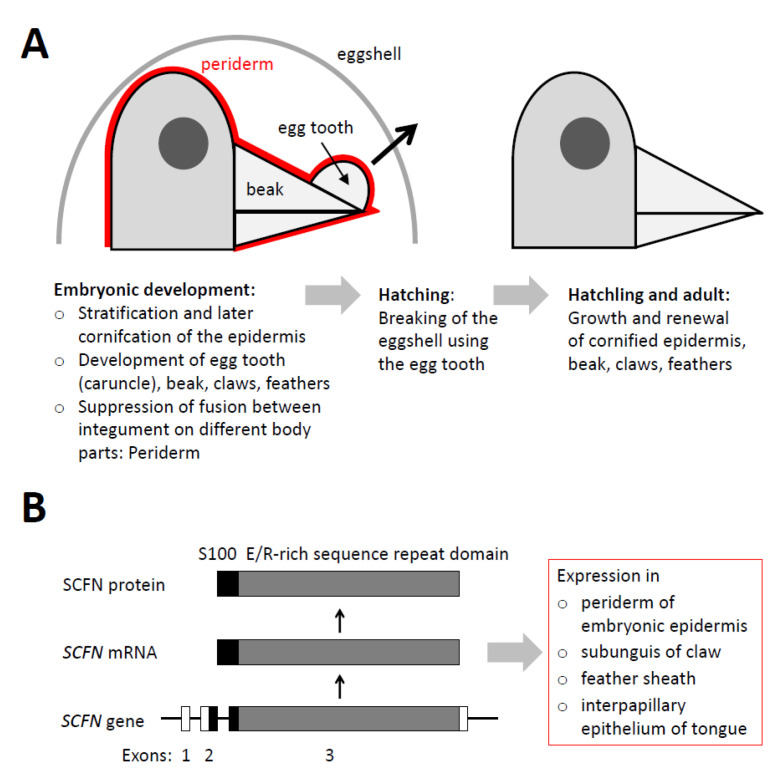
Schematic overview of beak development and SFTP gene expression. (**A**) Schematic depiction of the development and function of avian beak and egg tooth. (**B**) Organization and expression of the avian scaffoldin (SCFN)/trichohyalin-like gene, mRNA and protein. E, glutamic acid; R, arginine.

The embryonic development of birds and most reptiles occurs in eggs. Hatching is a crucial step in the life of these sauropsids and depends on the active breaking of the eggshell (Figure 1A). In squamate reptiles, a true tooth is used to open the egg [1,2], whereas in turtles, alligators and birds (together archelosaurs), a cornified carnuncle located on the upper beak or jaw is instrumental for hatching [3,4]. This caruncle is commonly referred to as the egg tooth, although it is not related to true teeth but rather consists of keratinocytes accumulating specific keratins and corneous β-proteins (CBPs), also known as β-keratins [5,6,7]. The caruncle-type egg tooth is the first cornified integumentary structure to appear during the embryonic development of birds [8]. Maturation of the egg tooth leads to its hardening which is completed before hatching. Later, the cornification of the rhamphotheca (beak) is completed and the egg tooth is lost (Figure 1A). Despite its critical role for the life of birds, the molecular control of egg tooth development in relation to development of the beak is only incompletely known [9].

The development of cornified skin appendages and the formation of the cornified layer of the epidermis depend on the differentiation of epidermal keratinocytes [10,11,12]. A gene cluster, known as the epidermal differentiation complex (EDC), plays crucial roles in the cornification of keratinocytes in mammals, reptiles and birds [13,14,15,16,17]. One class of EDC genes consists of one non-coding and one protein-coding exon. Examples include CBPs, epidermal differentiation cysteine-rich protein (EDCRP), epidermal differentiation protein starting with a MTF motif and rich in histidine (EDMTFH) and epidermal differentiation protein containing DPCC motifs (EDDM) which have been detected in feathers and other cornified skin appendages of birds and reptiles [17,18,19,20]. The other class of EDC genes is the S100 fused-type protein (SFTP) gene family which consists of one non-coding and two coding exons (Figure 1B).

SFTP genes give rise to proteins composed of a conserved N-terminal S100 domain, containing two calcium-binding EF-hand motifs, a central repeat domain of varying length and a protein-specific carboxy (C)-terminal domain. The C-terminal domain was suggested to contribute to the specific functions of each SFTP. In humans, seven SFTP proteins, filaggrin (FLG), filaggrin-2 (FLG2), trichohyalin (TCHH), trichohyalin-like1 (TCHHL1), hornerin (HRNR), repetin (RPTN) and cornulin (CRNN), have been identified. FLG, CRNN and TCHH have been best characterized [13,14]. FLG induces keratin filament aggregation in the upper layers of the epidermis, UV protection and hydration of the stratum corneum [21,22,23]. Polymorphisms in the *FLG* gene are associated with ichthyosis vulgaris and atopic dermatitis [24]. *CRNN* is expressed in the upper layers of the esophagus and epidermis as well as the inner root sheath of the hair follicle [25]. TCHH was also described as a keratin intermediate filament-associated protein that mechanically strengthens specific epithelia such as the inner root sheath of the hair follicle, the filiform papillae of the tongue or the nail isthmus [26,27]. Mutations of *TCHH* cause uncombable hair syndrome [28].

Recently, SFTPs were identified in birds, reptiles and amphibians [29,30], indicating that SFTPs are evolutionary ancient proteins required for terrestrial life. While turtles, geckos, alligators and chickens have two SFTP genes, *CRNN* and *Scaffoldin* (*SCFN*), also referred to as *Trichohyalin-like* [29,31], three SFTP genes, *CRNN*, *SCFN1* and *SCFN2*, are present in snakes [32] and only *SCFN* is present in the anole lizard [29,33], songbirds (Passeri), cormorants and Accipitriformes (eagles, hawks, vultures, secretary birds and relatives) [34]. Screening for SFTP expression sites in the chicken revealed CRNN or SCFN in the subunguis of claws, the feather sheath, epithelial cells located between papillae of the tongue and in the embryonic periderm (Figure 1B). The embryonic beak including the egg tooth yielded positive signals for SCFN [29] which prompted us to perform more detailed studies that are reported in the present manuscript.

Here, we investigate the expression of SFTPs during embryonic development of the beak and the egg tooth in chickens and quails. We report a conserved expression of SCFN in peridermal compartments of the upper and lower beak, suggesting a role in early development of the avian beak and egg tooth.

## 2. Materials and Methods

### 2.1. Animals

Tissue samples were prepared from chicken embryos and adult animals that were sacrificed in a previously published study [19]. Chicks were purchased from Schropper GmbH, Gloggnitz, Austria. Fertilized quail eggs were obtained from the Research Institute of Molecular Pathology, Vienna, Austria, in the course of a study published previously [35]. The eggs were incubated under standard conditions at 37.5 °C and 60–70% humidity. Pre-hatch eggs were removed at days 10, 14 or 18 (chicken) or 8, 10 or 14 (quail) of development. The shell of eggs was cut open to expose the embryo for tissues preparation.

All animal procedures were conducted according to the guidelines established by the Animal Care and Use Committee of the Medical University of Vienna. The Ethics Committee of the Medical University of Vienna decided that, in agreement with the national laws, a permission for sacrificing animals for organ preparation was not required.

### 2.2. RNA Extraction, cDNA Synthesis and Real-Time PCR

RNA was extracted from tissues that were homogenized with the Precellys system (VWR International, Radnor, PA, USA) using peqGOLD TriFast™ (VWR). Of note, RNA extraction from embryonic skin appendages was more efficient than extraction from fully cornified and hardened adult tissues. In brief, tissues were stored in RNAlater (Thermofisher, Vienna, Austria), cut into small pieces with sterile scalpels and incubated in 1 mL TriFast (VWR) on ice before and after homogenization. The tissue homogenizing Kit CK14 containing 1.4 mm ceramic beads (Precellys, VWR) was used for 2 treatments at 5500 rpm for 45 s. RNA was prepared with TriFast (VWR) and reverse-transcribed into cDNA using an iScript cDNA synthesis kit (Bio-Rad, Hercules, CA, USA) according to the manufacturers’ instructions. Real-time PCR was performed using the LightCycler^®^ technology and the LightCycler 480 SYBR Green I Master Kit (Roche Applied Science, Basel, Switzerland) according to the manufacturer’s protocol. *CRNN* cDNA of both chickens and quails was amplified with the following intron-spanning primer pair Crnn_f (5′-CAGGAGTTTGGGGATGTGAT-3′) and Crnn_r (5′-TTCTGGCTCCAGGTACTGCT-3′). Chicken *SCFN* and the house-keeping gene *HPRT1* were amplified with c-Scfn_f (5′-AGGAAGGCACAATCAACCAC-3′) and c-Scfn_r (5′-CACGACAAACCTCTGCTTCA-3′) and c-Hprt1-f (5′-AAAGTCATTGGTGGGGATGA-3′) and c-Hprt1-r (5′-GTAGTCGAGGGCGTATCCAA -3′. Quail *SCFN* and *GAPDH* [36] were amplified with the primers q-Scfn_f (5′-GGAAGATGGAGACCAATCCA-3′) and q-Scfn_r (5′-CAGGCCTTAGCCACTCTGAA-3′) and q-Gapdh_f (5′-CTAAGGCTGTGGGGAAGGTCA-3′) and q-Gapdh_r (5′- CATCAAAGGTGGAGGAATGGC-3′). The expression levels of *CRNN* and *SCFN* mRNA were normalized to the expression levels of housekeeping genes in each tissue, as described previously [37]. The relative expression levels in different tissues were defined as the ratio of expression in each tissue relative to that in an arbitrarily selected tissue.

### 2.3. Histological Staining and Immunohistochemistry

Tissues were fixed in formaldehyde and embedded in paraffin according to published protocols [29]. Hematoxylin and eosin staining was performed as reported previously [29]. The generation of the mouse anti-SCFN polyclonal serum directed against the peptide RYERTREDIAAEAE within the repeat unit in the C-terminal domain of chicken SCFN (Supplementary Figure S1A) was reported previously [29]. The epitope is conserved with the exception of 1 amino acid residue in SCFN of the quail (Supplementary Figure S1B,C). Immunohistochemistry with antiserum dilutions of 1:2000 (chicken) and 1:500 (quail) gave specific signals. The specific immunoreactivity was blocked by preincubation with antigenic peptide (4 μg peptide per 1 μL antiserum).

### 2.4. In Situ Hybridization

Tissue samples were fixed in formaldehyde and embedded in paraffin. There was no special treatment depending on the hardness of the samples. Hard cornified skin appendages were difficult to cut and to maintain in good shape during further processing, whereas samples from earlier stages of development were generally useful. The samples were sectioned at 5 µm thickness and mounted on Superfrost Ultra slides (Thermo Scientific). The sections were deparaffinized by melting at 58 °C for 1 h, followed by incubation in xylol for 2 × 15 min and an alcohol series (100%, 90%, 80%, 70%, 30% ethanol). After washing in phosphate-buffered saline (PBS) for 3 min, the sections were fixed in freshly prepared 4% paraformaldehyde in PBS on ice for 20 min. Then, the sections were washed with PBS for 2 × 3 min and incubated in 100 mM HCl at room temperature for 10 min. After washing with PBS for 2 × 3 min, the samples were incubated in 2× saline sodium citrate (SSC) at 70 °C for 30 min. Subsequently, the samples were washed with PBS for 2 × 3 min and digested with proteinase K (20 µg/mL in 50 mM Tris HCl) at 37 °C. After washing with PBS for 2 × 3 min, the samples were fixed with 4% paraformaldehyde in PBS on ice for 20 min, washed again with PBS and subjected to acetylation by 10-min incubation in 200 mL 0.1 M triethanolamine, pH 8, containing 500 µL acetic anhydride. Finally, the samples washed with PBS for 2 × 3 min, dehydrated in 30%, 70%, 80%, 90% and 100% ethanol and air-dried for 1 h. The slides were stored at −20 °C until hybridization with the probes.

As pretreatment before hybridization, the sections were incubated with hybridization solution at 55 °C for 1 h. A 30-mL stock of hybridization solution was made by mixing water and 15 mL deionized formamide (VWR), 6 mL 20× SSC (Sigma), 3 g dextran sulphate (Sigma, St. Louis, MO, USA), 0.3 mL 50× Denhardt’s solution (Sigma), 3 mL 10× sodium phosphate buffer, 75 µL denatured salmon sperm DNA (10 mg/mL, Life Technologies, Waltham, MA, USA) and 15 mg yeast t-RNA (Life Technologies).

Generation and validation of digoxigenin (DIG)-labeled sense and antisense probes for CRNN were reported previously [29]. DIG-labeled probes were denatured and mixed with hybridization solution at 80 °C and then incubated on ice for 10 min. Antisense and sense probes were applied to the pretreated sections at concentrations of 5 and 10 ng/mL, respectively, at a hybridization temperature of 55 °C [29]. After 20 h of incubation, the sections were washed with 2×SSC for 1 min, 3 times with 2×SSC + 50% formamide at 54 °C for 20 min, once with 2×SSC for 10 min and once with 0.2×SSC for 10 min. Subsequently, the DIG labels were detected immunochemically. The samples were incubated in a buffer containing TRIS (0.1 M) pH7.5, NaCl (1.15 M) and 2% bovine serum albumin (BSA) at room temperature for 15 min followed by incubation with anti-DIG-AP Fab-fragment (1:250, Roche) for 1 h. After washing in the aforementioned buffer for 2 times 5 min, samples were incubated with a buffer containing TRIS (0.1 M), NaCl (0.1 M) and MgCl_2_ (0.05 M), pH 9.5. Finally, nitro blue tetrazolium (NBT)/5-bromo-4-chloro-3-indolyl-phosphate (BCIP) (Roche) was added at a dilution of 20 µL/mL and the samples were incubated at room temperature in the dark until color development. Stainings obtained with the antisense probe were considered specific when the incubation of a serial section with the sense probe did not yield a staining signal.

## 3. Results

### 3.1. Avian SFTPs Are Expressed during Embryonic Beak Development of Chicken and Quail

**Figure 2:**
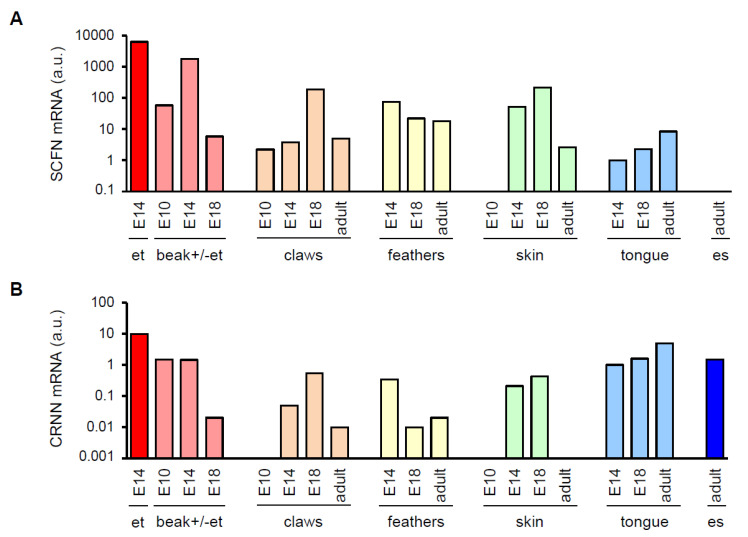
Expression of chicken SFTP mRNAs during embryonic development. RNA was extracted from indicated tissues of adult chicken and chicken embryos at developmental stages E10, E14 and E18. Following cDNA synthesis, real-time PCRs were performed. mRNA levels of *SCFN* (**A**) and *CRNN* (**B**) are shown in arbitrary units (a.u.) which were calculated by normalization to the housekeeping gene *HPRT1* in the same tissue and dividing the normalized value of each tissue by the normalized expression level in the tongue sample of stage E14. At developmental stage E14, the beak of the embryo was separated into two different parts—the egg tooth (et) and the remaining beak. es, esophagus.

**Figure 3:**
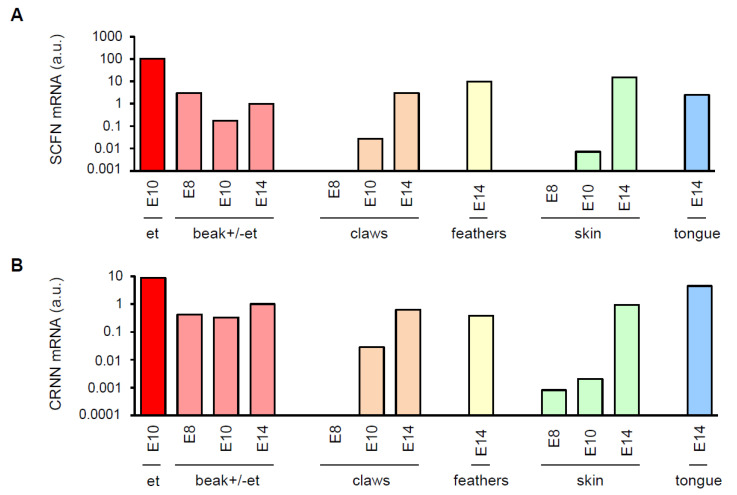
SFTP mRNAs expressed during embryonic development of quails. Indicated tissues were collected from quail embryos at developmental stages E8, E10 and E14, RNA was extracted, re-transcribed into cDNA and real-time PCRs were performed. mRNA levels of *SCFN* (**A**) and *CRNN* (**B**) are shown in arbitrary units (a.u.) which were calculated by normalization to the housekeeping gene *HPRT1* in the same tissue and dividing the normalized value of each tissue by the normalized expression level in the beak sample of stage E14. The RNA of the egg tooth (et) and the remaining beak of the E10 embryo was extracted separately.

To investigate the expression of the two SFTPs during avian embryonic development, we determined *SCFN* and *CRNN* mRNA levels in embryonic tissues of chickens and quails at different stages of development. *CRNN* and *SCFN* mRNAs were detected by RT-PCR in embryonic beak, skin, feathers and claws in both chickens and quails (Figure 2 and Figure 3). Although limited availability of samples did not allow statistical analysis of differences in this exploratory part of the study, high levels of *CRNN* and *SCFN* expression were consistently found in the egg tooth of both chickens (Figure 2) and quails (Figure 3).

**Figure 4:**
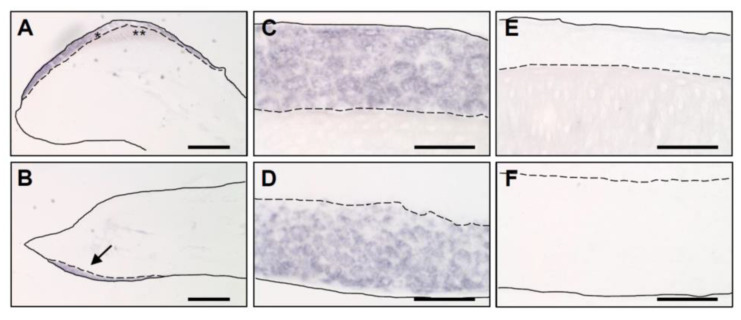
*CRNN* mRNA is expressed in the peridermal compartment of the chicken egg tooth. To localize *CRNN* mRNA-expressing tissues, an in situ hybridization of sections through the beak of chicken embryos at developmental stage E10 was performed. Pictures of the upper beak (**A**, higher magnification **C**) and lower beak (**B**, higher magnification **D**) are shown. The signal detected in the lower beak is indicated by an arrow (**B**). As negative control, the sense probe was applied to the upper (**E**) and lower beak (**F**). *, peridermal compartment of the egg tooth; **, cornified egg tooth. Bars: 400 µm (**A**,**B**), 40 µm (**C**–**F**).

To localize the expression of *CRNN*, we performed mRNA in situ hybridization in chicken embryos. *CRNN* mRNA was readily detectable in the multilayered periderm close to the tips of the upper and lower beak on embryonic day E10 of chicken development (Figure 4). Other parts of the beak where the keratinization and peridermal development was less progressed did not show *CRNN* labeling.

### 3.2. SCFN Forms Granules in Multilayered Periderm on the Beak

**Figure 5:**
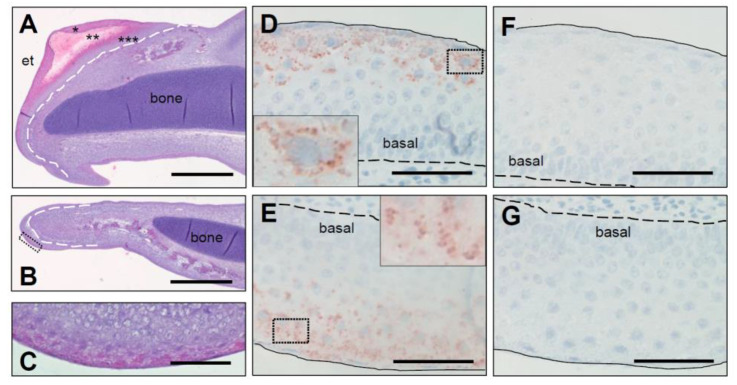
Expression of SCFN protein in the embryonic chicken egg tooth. Sections through the upper beak (**A**,**D**,**F**) and the lower beak (**B**,**C**,**E**,**G**) of chicken embryos at developmental stage E10 were immunohistologically stained with hematoxylin and eosin (**A**–**C**) or with mouse anti-SCFN serum (**D**,**E**). As control, anti-SCFN was blocked with the immunogen (**F**,**G**). The box in B indicates the area shown in a higher magnification in C. Insets in D and E show higher magnification of SCFN granules from the boxed areas of the sections. *, peridermal compartment of the egg tooth; **, cornified egg tooth; ***, beak underneath the egg tooth. et, egg tooth. Bars: 400 µm (**A**,**B**); 40 µm (**C**–**G**).

**Figure 6:**
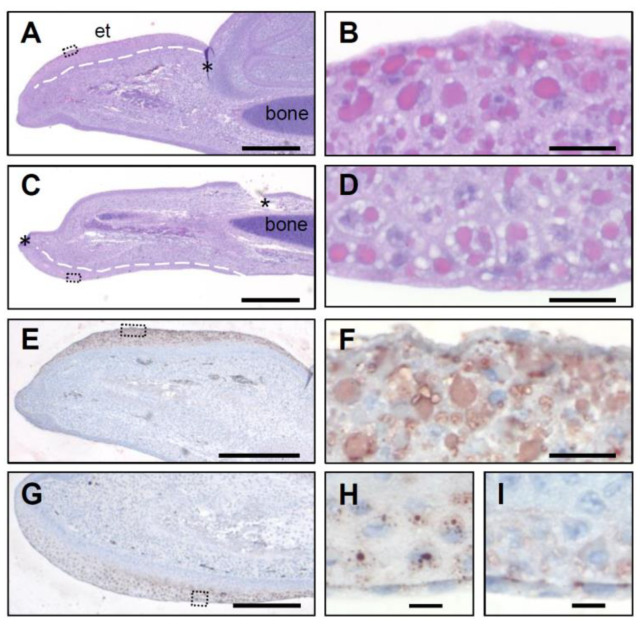
SCFN protein is expressed in the peridermal compartment of the quail egg tooth. Tissues were collected from quails at embryonic development stage E10. Hematoxylin and eosin stains of the upper (**A**,**B**) and lower beak (**C**,**D**) are shown. To localize SCFN protein in the quail beak, an immunohistochemical staining of SCFN was performed in the upper (**E**,**F**) and the lower quail beak (**G**,**H**). As control for the specificity of the result, anti-SCFN serum was blocked with the immunogenic peptide (**I**). Boxes in **A**, **C**, **E** and **G** indicate the region shown at higher magnification in **B**, **D**, **F** and **H**, respectively. Asterisks in **A** and **C** mark artefacts caused by the sectioning. Broken lines in **A** and **C** indicate border of periderm. et, egg tooth. Bars: 400 µm (**A**,**C**,**E**); 200 µm (**G**); 20 µm (**B**,**D**,**F**); 10 µm (**H**,**I**).

To determine the expression pattern of SCFN at the protein level, we used an antiserum that had been raised against an epitope of chicken SCFN [29]. This antiserum was predicted to cross-react with quail SCFN because of more than 90% sequence conservation of the epitope (Supplementary Figure S1). In the chicken, SCFN was detected in the multilayered periderm around the egg tooth (Figure 5D,E). It formed granules that corresponded in size and distribution to eosinophilic periderm granules [38] (Figure 5A–C), supporting the conclusion of a previous study that identified SCFN as a major component of granules in the periderm covering the embryonic epidermis [39]. A similar distribution of eosinophilic periderm granules (Figure 6A,B) and accumulation of SCFN were observed on the upper embryonic beak of quails (Figure 6E,F).

Histological investigation of the lower beak of chickens and quails revealed that the periderm was multilayered and contained eosinophilic granules similar to the pattern on the upper beak (Figure 5B,C and Figure 6C,D). Immunostaining demonstrated SCFN as a component of these granules in both species investigated (Figure 5E and Figure 6G–I). Preadsorption of the serum with the antigen blocked the immunostaining (Figure 5F,G), confirming its specificity. These data suggest that SCFN plays an evolutionarily conserved role in the development of the upper and lower beak in chickens and quails.

## 4. Discussion and Conclusions

The results of this study demonstrate that both CRNN and SCFN are expressed during the development of the integument of the avian beak. SCFN could be detected at the protein level in two species and consistently appeared in a granular pattern. Both SFTPs were expressed in the peridermal scaffold of the forming egg tooth but also on the lower beak. According to a comprehensive review article by Clark [40], an egg tooth-like structure is formed on the lower beak of diverse species of birds, such as the cormorant (*Phalacrocorax auritus*), red kite (*Milvus milvus*), common moorhen (*Gallinula chloropus*), southern black korhaan (*Afrotis afra*), northern lapwing (*Vanellus vanellus*), black-tailed godwit (*Limosa limosa*), pied avocet (*Recurvirostra avosetta*) and mourning dove (*Zenaida macroura*), and an egg tooth anlage is present on the lower beak of the chick. Therefore, it is likely that the accumulation of peridermal layers and the expression of SCFN during development of the lower beak correspond to the growth of an egg tooth-like structure which, however, needs further characterization.

As our immunohistochemical data suggest that a modification of the periderm supports the development of the egg tooth on the beak, this raises the question as to how these integumentary structures have evolved. The primordial and evolutionary ancient function of the periderm is to prevent the fusion between integumentary epithelia of different body parts [41]. The fact that a periderm lines the skin and the oral epithelia of fish [42] indicates that it has evolved prior to the water-to-land transition of tetrapods. In avian embryos, a one- or two-layered periderm covers the entire integument, including the beak [43], and SCFN and CRNN are expressed in this thin “conventional” periderm [29]. We propose that the multilayered periderm around the egg tooth has evolved from the normal thin periderm and the peridermal functions of SFTPs were maintained there.

Recently, it was suggested that the presence of a caruncle-type egg tooth in oviparous tetrapods allowed the repeated evolutionary loss of real teeth, i.e., edentulism, and the evolution of rhamphothecae (beaks) in turtles and birds [8]. Indeed, the existence of an egg tooth in beak-less tetrapods, such as crocodilians, supports the notion that the egg tooth is evolutionary older than the beak. It will be interesting to investigate whether the periderm also forms multiple layers around the developing egg tooth in turtles and crocodilians.

A recent large-scale study of genome sequences showed conservation of *SCFN* in birds, although the extremely long third exon of *SCFN* has not been completely or unambiguously sequenced in all bird genome sequences currently available [34]. By contrast, CRNN is absent or disrupted by mutations in songbirds and at least two other clades of birds, suggesting that the functions of CRNN are dispensable in these evolutionary lineages [34]. Accordingly, the expression of CRNN in the beak and egg tooth apparatus, though conserved in chickens and quails, may be functionally redundant with that of SCFN.

The protein features of SCFN are trichohyalin-like [26] with regard to the presence of a long C-terminal domain that is rich in glutamic acid (E) and arginine (R) which together constitute close to half of the total amino acid content of these proteins [29]. The presence of a double band on Western blots [29] indicates post-translational modification. The latter may change the isoelectric point (pI 5.3 predicted for chicken SCFN, GenBank accession number NP_001338424.2) and affect both interactions with other proteins and the affinity for histological dyes. Our results suggest that SCFN-positive periderm granules correspond to eosinophilic granules visible in hematoxylin and eosin stainings of the embryonic beak (Figure 5B,C and Figure 6C,D). Besides modifications of SCFN, the presence of other proteins may influence the staining properties of these granules.

SFTPs were reported to interact with keratins, undergo cross-linking by transglutamination, serve as precursors for functional peptides and amino acids and contribute to degradation of the nucleus [21,22,27,44,45]. However, the relative importance of possible mechanisms of action are not known for mammalian SFTPs and even more so for avian SFTPs. The association of *SCFN* expression with important integumentary processes, such as development of the beak and egg tooth, indicates that *SCFN* should be a prime candidate for future studies of reverse genetics in birds.

## Data Availability

Data are contained within the article or Supplementary Material.

## Supplementary Materials

The following are available online at https://www.mdpi.com/2073-4425/12/2/248/s1, Figure S1: Sequences of S100 fused-type proteins (SFTPs) in chickens and quails.
