# Supplementary material for: The Trichohyalin-Like Protein Scaffoldin Is Expressed in the Multilayered Periderm during Development of Avian Beak and Egg Tooth
Source: Genes (Basel). 2021 Feb 10;12(2):248. doi: 10.3390/genes12020248 (PMC7916365; doi:10.3390/genes12020248)
Supplement: Supplementary file 1 [file genes-12-00248-s001.zip › Supplement.pdf]

S100 fused-type proteins (SFTPs) in chicken and quail

A) Accession numbers and amino acid sequences of chicken SFTPs

>NP_001165847.1 Chicken_cornulin (CRNN)
MAQLQENINGIITVFYTYARSDGDSSTLSRGELRQLIEQEFGDVITDARDPRTVDEVLFFLDEDSSGKIDFGEFLSLIFRVAKACHR
QLQQYLEPEYDQELTVQEEADGEQQHNQVLEQGVSEQVQEGGTPQQDQDTQQHQESEEPKNHQGTQQKQEGGTPKYQDTQKIEKSEI
PKVQDTQKTQEVETPKNNESTQQKKSETPKDNQDTHQDDRAKTPEQSPKRGENVVTKSPEEDRNTQEAEKTPKHDPKPHQDKVTETP
KLGQDHHQRHELKPAEQSHTSPTKTPGGTTDKTKDHVAHWQDPSPKGTQELPPPVRGTSPHPDPRPSGTQHDPAHLPNPTVIVREGS
GAEAEHVPGQQQHGAHGKGKHVAEQEHLQPQWPPRK

>NP_001338424.2 Chicken_scaffoldin (SCFN)
MSHFLDSVSTIITVFYQHAKEDGDQSKLNRRKMKEFIEKEFADAIVNPHDPQTIEKILQFLEWDGDGEIDFNEFLLLVFRVAKACYW
YLQKGQCLLQRTKLITSSKTIQEPEIKNRGSRQQLQEEEPQTLERNRHPPCIPEPQPDTRVQDLETHEETGSHRQQHNTQSRADARQ
STRPSEIIPQEYEEQTQEPCDQRSRQRRQPPELDRLGDLQSHKHGSLKAAQNDERQNQEKPQREQLADVRSCSHSCEPQLLPDRRSD
RQPREPALPAYDQRNHRPQDQEAVAHGRSSRRPHEPEEADRGRHNQPRNPELLKDERSRNHLRKLEQKELERSSRQPCKPECLDSER
LHQSYLQEPLVISHKYNNTRELEEDYYEQGKTQKDRKSCESMMEERTQEDSVAEAEATVKIRRETQKREREEEAKRPRESARYQRTQ
ENTVAEAEADVEIHRVSRGRERELEDRRPHESVRHVKTQENIVAEAEADVEIRRVSREREREQEDRRPRDSERYDRTREDIVAEAEA
NVEIRRVSRGREREEDRRPDELVRYVKTQEDIVAEAEADVQIPCVSRKREREGELRRPRESVRYERTREDIAAEAEADVEIRHVTQK
SETGQEDRKPRESQRYDKTREDVVAEAEADVEIRRVSRGREREQEDRRPRESQRYERTREDIAAEAEADVEIRRVSRERKREEEARR
PRELLRSGQRWEDIVAEAEADVEIHRVSREREREEEARRPRDSVRYERTREDIVAEAEADVEIRRVSRGREREQEDRRPRDSVRYER
TREDIVAEAEADVEIRHVSRGREREQEARRPRDSVRYERTREDIVAEAEADVEIRRVSRGREREEARRPHELVRYVKTQEDLVAEAE
ANVEIHRVSREREREEEARRPRDSVRYERTREDIAAEAEADVEIRRVSRGREREEARRPNELVRYVKTQEDTVAEAEADVEIHHVSR
EREREEEARRPRDSVRYERTREDIVAEAEADVEIRRVSRGREREQEDRRPRESQRYERTREDIAAEAEANVEIRRVSRERKREEEAR
RPRDSVRYERTREDTVAEAEADVEIRRVSRGREREEAKRPCELVRYVKTQEDIVAEAEADVEIHRVSRGREREQDNRRPRESQRYER
TREDIVAEAEAEVEIHRASRGREREEARRPDDSVRYVKTREDIVAEAEAEVEIRRVSREREREEEARRPRDSVRYERTREDIVAEAE
ADVEIRRVSRGREREQEDRRPRESQRYERTREDIVAEAEADVEIRHVSREREREEEARRPRESVRYERTREDIVAEADLEIRRVSRE
RKREEEARRPRESQRYERTREDIVAEAEADVEIRRVSRGREREEARRPRESVRYVKTQEDIVAEAEADVEIYRVSREREREEARRPR
DSVRYDRTREDIAAEAEADVEIRRVSRGREREEARRPNELVRYVKTQEDTVAEAEADVEIHRVSREREREEEARRPRDSVRYERTRE
DIVAEAEADVEIRRVSRGREREQEDRRPRESQRYERTQEDIVAEAEADVEIRRVSRGREREQEDRRPHKSQRYERTREDIAAEAEAD
VEIRRVSRGREREQEDRRPRESQRYERTREDIVAEAEADVEIRRVSRGREREEARRPRELVRYVKTQEDIVAEAEADVEIHRVSRER
EREEEARRPRDSVRYERTREDIVAEAEADVEIRRVSQGREREQEDRRPCESQRYERTREDIVAEAEADVEIRRVSRGREREQEDRRP
CESQRYERTREDIVAEAEAEVEIHRASRGREREEARRPDDSVTYVKTREDIVAEAEAEVEIRRVSREWEREEEARRPRDSVRYERTR
EDIVAEAEAEVEIHRASRGREREEARRPDDSVRYVKTREDIVAEAEAEVEIRRVSREREREEEARRPRDSVRYERTREDIVAEAEAD
VEIRRVSQGREREQEDRRPCESQRYERTREDIVAEAEADVEIRRVSRGREREQEDRRPCESQRYERTREDIVAEAEAEVEIHRASRG
REREEARRPDDSVRYVKTREDIVAEAEAEVEIRRVSREWEREEEARRPRDSVRYERTREDIVAEAEAEVEIHRASRGREREEARRPD
DSVRYVKTREDIVAEAEAEVEIRRVSREREREEEARRPRDSVRYERTREDIVAEADLEIRRVSRERKREEEARRPRESQRYERTRED
IVAEAEADVEIRRVSRGREREEARRPRESVRYVKTQEDIVAEAEADVEIYRVSREREREEARRPRDSVRYDRTREDIAAEAEADVEI
RRVSRGREREEARRPNELVRYVKIQEDTVAEAEADVEIHRVSREREREEEARRPRDSVRYERTREDIVAEAEADVEIHRVSRGRERE
QEDRRPRESQRYERTQEDIVAEAEADVEIRRVSRGREREQEDRRPHKSQRYERTREDIAAEAEVDVEIRRVSRGREREQEDRRPRES
QRYERTREDIVAEAEADVEIRRVSRGREREEARRPRELVRYVKTQEDIVAEAEADVEIHRVSREREREEEARRPRDSVRYERTREDI
VAEAEADVEIHRVSREREREEEARRPRDSVRYERTREDIVAEAEAEVEIRRVSQGREREQEDRRPCESQRYERTREDIVAEAEADVE
NRRVSRGREREQDDRRPRESQRYERTREDIVAEAEAEVEIHRASRGREREEARRPDDSVRYVKTREDIVAEAEAEVEIRRVSRERER
EEEARRPRDSVRYERTREDIVAEAEADVEIRRVSRGREREQEDRRPRESQRYERTREDIVAEAEADVEIRHVSREREREEEARRPRD
SVRYERTREDIVAEADLEIRRVSRERKREEEARRPRESQRYERTREDIVAEAEADVEIRRVSRGREREEARRPRESVRYVKTQEDIV
AEAEADVEIYRVSREREREEARRPRDSVRYDRTREDIAAEAEADVEIRRVSRGREREEARRPNELVRYVKIQEDTVAEAEADVEIHR
VSREREREEEARRPRDSVRYERTREDIVAEAEADVEIRRVSRGREREQEDRRPRESQRYERTQEDIVAEAEADVEIRRVSRGREREQ
EDRRPHKSQRYERTREDIAAEAEVDVEIRRVSRGREREQEDRRPRESQRYERTREDIVAEAEADVEIRRVSRGREREEARRPRELVR
YVKTQEDIVAEAEADVEIHRVSREREREEEARRPRDSVRYERTREDIVAEAEADVEIHRVSREREREEEARRPRDSVRYERTREDIV
AEAEADVEIRRVSQGREREQEDRRPCESQRYERTREDIVAEAEADVEIRRVSRGREREQDDRRPRESQRYERTREDIVAEAEAEVEI
HRASRGREREEARRPDDSVRYVKTREDIVAEAEAEVEIRRVSREREREEEARRPRDSVRYERTREDIVAEAEADVEIRRVSRGRERE
QEDRRPRESQRYERTREDIVAEAEADVEIRHVSREREREEEARRPRDSVRYERTREDIVAEAEADVEIRRVSRGREREQEDRRPRES
QRYERTQEDIVAEAEADVEIRRVSRGREREQEDRRPHKSQRYERTREDIAAEAEADVEIRRVSRGREREQEDRRPRESQRCERTRED
IVAEAEADVEIRRVSRGREREEARRPRELVRYVKTQEDIVAEAEADVEIHRVSREREREEEARRPRDSVRYDRTREDIVAEAEADVE
IRRVSQERERQEEARRPRDSVRYDRTREDIVAEAEADVEIRHVTQKREREQEDRRPRKSQRYERTREDIVAEAEAEVEIRRVSRGRE
REEARRPNDSVRYVKTREDIVAEAEAEVEIRRVSREREREEEARRPRDSVRYERTREDIVAEAEADVETRRVSRGREREQEDRRPHE
SQRYERTREDIVAEAEADVQIHRVSQKREREQEARRPRESVRYEKTQEDILAEAEADVEIRHVSREREREEARRPRESLRYERTQED
IVTDAEAEVCREEVERADSLHEYEEAVKERRAVRARAREEAPVRERRLDRQRDLEVERNRFPGHDDQGERQERSLYQTVDVDSRDLV
PPGEVPSVTDVRVRYIPSDPDVRPEVQAVPEPCEPQSIAYLIHVIQNLNDPKATTYEIICQQSNDSGQPVYVRKCYVSPQPPVGPCK
ESSVLEPELQHLPSSSSQENARELEEPRKGEIRERSATGVKEGASAPELDSLEDDTSQAETREDRRDHQSPSVHDVEEKNHQPEGDG
PKATEEEVSQEAESQGNKDREAKSRSPAARAPELQEAHKQGQQAAEEPCHPVPQRDEAGCCHEDAELPSPDRSRQAREEENSKRSSQ
QENNTQPQREEERSPMAPQALSHPMKDEAKAP

Antibody binding sites are shaded yellow. Additional putative binding sites with around 90% sequence conservation are present but not highlighted.

B) Amino acid sequences of SFTPs and accession numbers of the coding sequences in the genome of the Japanese quail

```
>Quail_cornulin (CRNN)
MAQLQENINGIITAFYTYARSDGDCSTLSRGELRQLIEQEFEDVIVDARDPRTVEKVLLFLDEDSSGKVDFGEFLSLVFRVAKACHR
QFQQYLEPEDDQELTVQEEADGEQHHNQVQEQGVSEEVQESGTTQQHQEGEEEKNHQDTQQKQEGETPKNQDTQNIEKTETPKVQDT
QQTQEVETPNNQGTQEKKIEIPKDIQDTHQDDRATAPEEGPKRGENVVTETPEKDRNTPEAEKTPKHDPKLHQDKVTETPELGQNNH
HREELEPAEQSHTSPTVTPGRTTDTNKNPSPKETQELPPPVRGTDPHPDPQPSGTRRDPAHPPDLTVTVREGSVAEAERVPGHQQHG
AHGKGKHVVEKENLQPQRPPRK
```

Coding sequence (GenBank accession number NC_029540.1, nucleotide numbers 891426-891563, 890345-891316, reverse complement)

```
>Quail_scaffoldin (SCFN) preliminary*
MSHFLDSVSTIITVFYQHAKEDGDQSKLNRRKMKEFIQKEFADAIANPHDPQTIDKILQFLEWDGDGEIDFNEFLLLVFRVAKACYW
YLQRGQCILQRTKLITSSKTIREPEIKNRGSRQQLQEEEPQTHPPYIPEPQQDTRVQDLEIREETGSHRQQRNTESRADARQSSRPR
EMIPQEYEEQSQEPCDQGSSRRRQPPELDRLGDLQYHKRGSLKAAQRDERQNLREQLADVRSCSHSCEPQLLPDRRSGRQPREPTLP
AYDQRNHRPQDQEAVAQARGSRRPHEPEEADRGRRNQPRKPELLKDERSRNHLRELEQKELERSSRQPCKPECLDSEWQHQSYLQEP
LVIDHRYNKTREMEDYYEQGKIQKDRRPRESMTYERTEEDIVVEAEADVEIRRETQKRERGLEARRPHESVKYVKTREDIEAEAEAD
VEIHRVSRVREREEEARRPGESVRRERTREDIVAELEGDVEIRRVTQERKRVVEARRPDESARYERTREDIVAEAEVDVDIRRVTRT
REREDTRRPRESVRYERTREDIVAEAEADVEIHRVSRVREREEEPRRPRESVRRERTREDIVAELEGDVEIRRVTQERKRKEEARRP
GEPDRYRRTREDIVAEAEADVEIHHVTRKREREDARRPHESVRYERTREDIVAEAEADVEISRVSREVERAEQARRPGESVRRERTR
EDIVAEVEGDVKIHRVTQECKRVVEARRPDASARYERTREDIVAEAEVDVDIRRVSRVREREEEPRRPRESVRRERTQEDIVAEVEG
DVEIHRVTRKTGERGCQETQRESVRYERRWRHCG
```

Antibody binding sites are shaded yellow.

Coding sequence of SCFN gene
NC_029540.1, nucleotides 885529-885666, 882990-885305 (reverse complement)

* Because of possible sequence assembly errors in the repetitive sequence region, the sequence is considered preliminary. The coding sequence may actually be longer and include sequences up to position NC_029540.1, nucleotide 878530 (reverse complement).

C) Alignment of binding sites of the anti-scaffoldin antiserum

```
Chicken   RYERTREDIAAEAE
Quail     RYERTREDIVAEAE
```

Supplementary Figure S1. Sequences of S100 fused-type proteins (SFTPs) in chicken and quail. The amino acid sequences of chicken cornulin (CRNN) and scaffoldin (SCFN) were downloaded using the indicated accession numbers from GenBank (**A**). Sequences of quail CRNN and SCFN were predicted on the basis of the current genome sequence of the Japanese quail (**B**). Binding sites of the anti-scaffoldin antiserum (Mlitz et al. 2014) are highlighted by yellow shading in SCFN sequences and compared between chicken and quail (**C**).
